# Potentiation of Antibiofilm Activity of Amphotericin B by Superoxide Dismutase Inhibition

**DOI:** 10.1155/2013/704654

**Published:** 2013-09-01

**Authors:** Katrijn De Brucker, Anna Bink, Els Meert, Bruno P. A. Cammue, Karin Thevissen

**Affiliations:** ^1^Centre of Microbial and Plant Genetics, KU, Leuven, 3001 Heverlee, Belgium; ^2^CMPG, Kasteelpark Arenberg 20, 3001 Heverlee, Belgium

## Abstract

This study demonstrates a role for superoxide dismutases (Sods) in governing tolerance of *Candida albicans* biofilms to amphotericin B (AmB). Coincubation of *C. albicans* biofilms with AmB and the Sod inhibitors N,N′-diethyldithiocarbamate (DDC) or ammonium tetrathiomolybdate (ATM) resulted in reduced viable biofilm cells and increased intracellular reactive oxygen species levels as compared to incubation of biofilm cells with AmB, DDC, or ATM alone. Hence, Sod inhibitors can be used to potentiate the activity of AmB against *C. albicans* biofilms.

## 1. Introduction

*Candida albicans* biofilms are responsible for device-related infections in most nosocomial diseases [1]. Such infections are particularly serious because biofilm-associated *Candida* cells are relatively resistant to a wide spectrum of antifungal drugs, including amphotericin B (AmB) [2]. The cause of this increased resistance is not yet fully elucidated but could be due to a combined action of different mechanisms including (i) expression of resistance genes, (ii) drug binding to the extracellular matrix, (iii) the change in membrane composition, or (iv) the presence of persister cells, which are cells that can survive high doses of an antimicrobial agent [3]. Due to this increased resistance, biofilm eradication and treatment of associated infections are challenging. The recalcitrance to antifungal therapy remains the biggest threat to patients with fungal biofilms and is an increasingly significant clinical problem [4]. Understanding the role of fungal biofilms during infection should help the clinical management of these recalcitrant infections. Until now, no vaccines are available to combat fungal infections, despite the considerable growth in the research field [5]. Therefore, the use of antimycotics is currently the only clinical solution for these infections. Among the current antimycotics in clinical use, only the liposomal formula of AmB and echinocandins has shown consistent *in vitro* and *in vivo* activity against *C. albicans* biofilms [6–8]. AmB is a fungicidal polyene and, apart from its interaction with ergosterol and subsequent pore formation, induces accumulation of reactive oxygen species (ROS) and apoptosis in planktonic and biofilm *C. albicans* cells [9, 10]. Despite its high efficacy as an antimycotic, the effective concentrations of AmB required for elimination of *Candida* biofilms are often hepatoxic and/or nephrotoxic [11, 12]. Therefore, in order to improve the potential of AmB for treatment of such biofilms, it is recommended to search for new approaches in which the effective concentration of AmB against *C. albicans* biofilms and consequently also its negative side effects are reduced. 

In this study, we aimed at identifying compounds that lead to increased antibiofilm activity of AmB. Recently, we reported that superoxide dismutases (Sods) are involved in *C. albicans* biofilm persistence to the ROS-inducing antifungal miconazole. *C. albicans *contains 6 different Sods, which are involved in the detoxification of ROS by converting O_2_^−^ into molecular oxygen and hydrogen peroxide [13, 14]. Sod1, Sod4, Sod5, and Sod6 of *C. albicans* are Cu,Zn-containing superoxide dismutases [14] that can be inhibited using the Cu,Zn-Sod inhibitor N,N′-diethyldithiocarbamate (DDC), which chelates copper [15]. We previously demonstrated that this inhibitor potentiates the activity of miconazole against *C. albicans* persister cells within biofilms, thereby allowing ROS build-up and intensive killing of the persister cells [16]. Ammonium tetramolybdate (ATM) is another copper chelator which is used in clinical applications. For example, ATM is used therapeutically in the treatment of copper metabolism disorders (e.g., Wilson's disease) where it reduces copper adsorption or removes excess copper from the body [17–19]. ATM inhibits activities of a variety of Cu-utilizing enzymes, including Cu,Zn-Sod1 [20–22]. In the present study, we investigated a putative effect of DDC or ATM on the activity of AmB against *C. albicans *biofilms and planktonic cells.

## 2. Materials and Methods

### 2.1. Materials, Yeast Strains, Plasmids, and Growth Media

*C. albicans* CA-IF100 [13], *C. albicans* clinical isolates F17, G6 [23], and 2CA [16] were used in this study. Growth medium was YPD (1% yeast extract, 2% peptone, and 2% glucose) and SC (1% CSM, complete amino acid supplement mixture, 1% YNB, yeast nitrogen base; 2% glucose). N-N′-diethyldithiocarbamate (DDC) (stock = 1 M in water), ammonium tetrathiomolybdate (ATM) (stock = 1 M in DMSO) and AmB (stock = 5 mM in DMSO), were purchased from Sigma (St. Louis, MO, USA). DHE was purchased from Life technologies (Paisley, UK). Phosphate-buffered saline (PBS) was prepared by combining 8 gl^−1^ NaCl, 0.2 gl^−1^ KCl, 1.44 gl^−1^ Na_2_HPO_4_, and 0.24 gl^−1^ KH_2_PO_4_ (pH 7.4). 

### 2.2. Drug Susceptibility Testing against Planktonic *C. albicans *Cells

Overnight cultures of* C. albicans* were washed in PBS and diluted in SC medium to 1 × 10^6^ cells/mL. Cultures were treated with 0.156 *μ*M AmB, 10 mM DDC or 0.156 *μ*M AmB, and 10 mM DDC and incubated for 24 hours at 37°C. DMSO (2%) was used as a control treatment. Next, cells were diluted in PBS and plated on YPD agar plates. Afterwards, the number of colony-forming units was determined and the percentage of surviving *C. albicans *cells was calculated relative to the DMSO control treatment.

### 2.3. Drug Susceptibility Testing on *C. albicans* Biofilms

The activity of AmB (final DMSO concentration = 2%) in the absence or presence of 10 mM DDC or 10 mM ATM against 16 h old *C. albicans *biofilms was assessed in PBS as described previously [16]. DMSO (2%) was used as a control treatment. Briefly, after incubation for 24 h, biofilms were washed, resuspended in PBS by vigorous vortexing, and plated on YPD agar plates. The fraction of viable biofilm cells was determined by counting the colonies and calculating the percentage of surviving *Candida *cells, relative to the control treatment. 

### 2.4. ROS Accumulation Assay in *C. albicans* Biofilm Cells

Quantification of ROS using 2′,7′-dichlorodihydrofluorescein diacetate (DCFHDA) was performed as previously described [16]. Quantification of ROS was additionally determined using dihydroethidium (DHE). To this end, *C. albicans *biofilms were treated with 1 *μ*M AmB in presence or absence of 10 mM DDC or 10 mM ATM (final DMSO concentration = 2%). As a control treatment, 2% DMSO was used. After 24 h incubation at 37°C, biofilms were washed and resuspended in PBS by vigorous vortexing. A sample was taken for colony counting, after which the biofilm cells were incubated for 20 minutes at 37°C with 20 *μ*M DHE. After washing, fluorescence was measured (510 nm/595 nm) using a fluorescence spectrometer and values were normalized to the number of CFUs. 

### 2.5. Statistical Analysis

Statistical analysis was performed using unpaired *t*-test. Differences were considered significant if *P < 0.05; **P < 0.01; ***P < 0.001. Data of all experiments are represented by the mean ± SEM.

## 3. Results and Discussion

### 3.1. DDC Increases the Antibiofilm Activity of AmB against *C. albicans*

**Figure 1:**
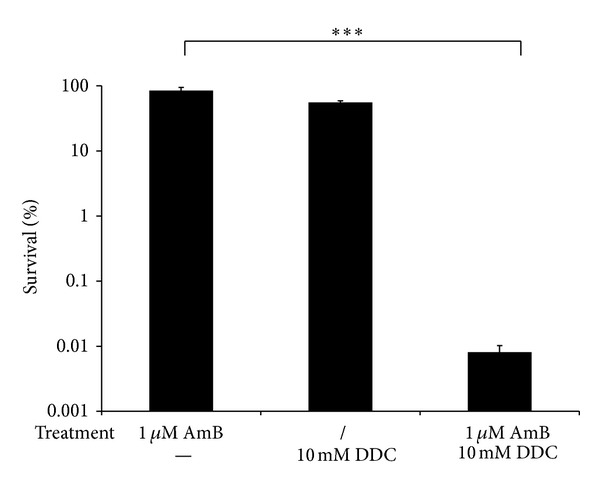
Effect of N-N′-diethyldithiocarbamate (DDC) on AmB-tolerant cells in *C. albicans* CA-IF100 biofilms. Biofilms were treated with or without (/) 1 *μ*M AmB in presence or absence (−) of 10 mM DDC. After 24 hours, biofilms were washed with PBS and the percentage survival of *C. albicans *cells, relative to the control treatment (2% DMSO), was determined by plating the biofilm cells on YPD plates. Data represent the mean and SEM for one representative experiment out of two, each consisting of triplicate measurements. ***P < 0.001.

First we investigated the effect of N-N′-diethyldithiocarbamate (DDC) on the activity of Amphotericin B (AmB) against *C. albicans* CA_IF-100 biofilms. To this end, a concentration of AmB that had no significant effect on the viability of *C. albicans* biofilm cells was used. Treatment of *C. albicans* biofilms with 1 *μ*M AmB did not result in a statistically significant reduction of viable biofilm cells compared to control treatment (Figure 1). We used these concentrations of AmB to further investigate the potential of DDC on potentiating the antibiofilm activity of AmB against *C. albicans* biofilms. Since Lushchak and colleagues previously demonstrated that treatment of *Saccharomyces cerevisiae* with DDC caused a dose-dependent inhibition of Sod activity *in vivo*, with 75% inhibition occurring at 10 mM DDC [15], we used a similar concentration in our experiments. Coincubation of *C. albicans *biofilms with 1 *μ*M AmB and 10 mM DDC resulted in an approximately 10,000-fold reduction of viable biofilm cells as compared to AmB or DDC treatment alone. More specifically, treatment of *C. albicans* biofilms with 1 *μ*M AmB and 10 mM DDC resulted in only 0.008 ± 0.002% viable biofilm cells (*P* < 0.001), whereas treatment of biofilms with 1 *μ*M AmB alone resulted in 84.80 ± 9.66% viable biofilm cells. Treatment of *C. albicans* biofilms with 10 mM DDC alone resulted only in a 2-fold reduction of the viable biofilm cells (*P* < 0.05) (Figure 1), pointing to a clearly enhanced antibiofilm activity of AmB when combined with DDC.

### 3.2. The Antibiofilm Activity of AmB against *C. albicans* Clinical Isolates is Enhanced by DDC

**Figure 2:**
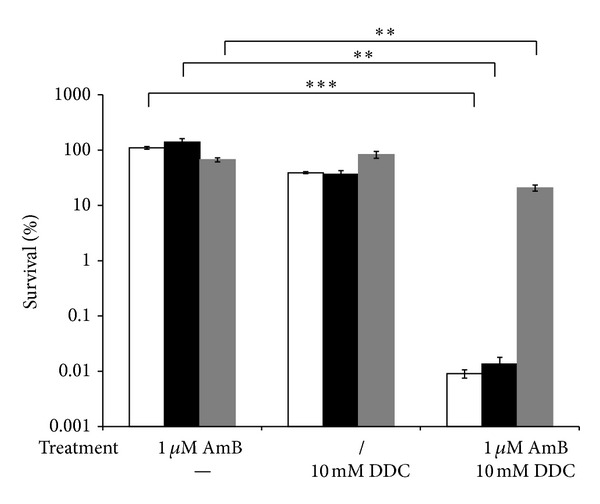
Effect of DDC on AmB-tolerant cells in biofilms of *C. albicans* clinical isolates F17, G6, and 2CA. *C. albicans* biofilms of F17, G6, or 2CA were treated with or without (/) 1 *μ*M AmB in presence or absence (−) of 10 mM DDC. Afterwards, biofilms were washed with PBS and survival of *Candida *cells was determined by plating the biofilm cells on YPD plates. The AmB-tolerant fraction in presence or absence of 10 mM DDC was determined relative to DMSO treatment. Data represent the mean and SEM for one representative experiment out of two, each consisting of triplicate measurements. F16 (white bars), G6 (black bars), 2CA (grey bars). **P < 0.01; ***P < 0.001.

To evaluate the above findings further, we assessed the antibiofilm activity of AmB and DDC using 3 *C. albicans* clinical isolates. Clinical isolates F17 and G6 are characterized by increased levels of AmB-tolerant persisters [23] whereas strain 2CA was isolated from the voice prosthesis of different laryngectomized patients [16]. The percentage of viable biofilm cells upon treatment of *C. albicans* F17, G6, or 2CA biofilms is represented in Figure 2. Coincubation of F17 or G6 biofilms with 1 *μ*M AmB and 10 mM DDC resulted in an approximately 10,000-fold reduction of viable biofilm cells as compared to AmB treatment alone. More specifically, treatment of biofilms of F17 or G6 with 1 *μ*M AmB and 10 mM DDC resulted in only 0.009 ± 0.002% (*P* < 0.001) or 0.01 ± 0.004% (*P* < 0.01) of viable biofilm cells, respectively, whereas treatment of biofilms of F17 or G6 with 1 *μ*M AmB alone had no effect on the viability of these biofilm cells. Treatment of these biofilms with 10 mM DDC alone resulted in a 3-fold reduction of viable biofilm cells (*P* < 0.05) (Figure 2). Treatment of 2CA biofilms with 1 *μ*M AmB and 10 mM DDC resulted in an approximately 3-fold reduction of viable cells compared to AmB treatment alone. More specifically, coincubated 2CA biofilms resulted in 20.6 ± 2.6% (*P* < 0.01) of viable cells, whereas treatment of these biofilms with 1 *μ*M AmB alone resulted in 66.8 ± 5.7% of viable cells. Treatment of 2CA biofilms with 10 mM DDC alone resulted in 82.7 ± 11.5% viable cells (Figure 2). These results confirm that inhibition of Sod activity by DDC can potentiate the antibiofilm activity of AmB against various *C. albicans* clinical isolates. The extent of potentiation of the AmB antibiofilm activity seems strain dependent.

### 3.3. Potentation of Antifungal Activity of AmB by DDC is Not Biofilm Specific

**Figure 3:**
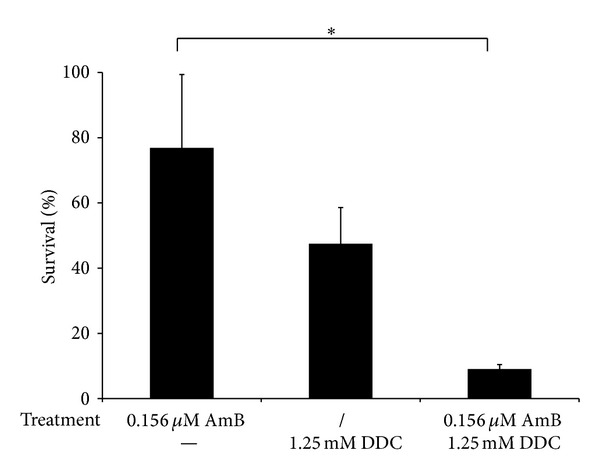
Effect of combined treatment of DDC and AmB on planktonic *C. albicans *cell cultures. Planktonic *C. albicans *cultures were treated with or without (/) 0.156 *μ*M AmB in presence of absence (−) of 1.25 mM DDC for 24 hours and afterwards plated on YPD plates. The percentage survival relative to the control treatment (2% DMSO) is shown. Data represent the mean of 2 independent biological experiments, each consisting of two measurements. *P < 0.05.

The activity of AmB in the presence of DDC against planktonic *C. albicans *CA_IF-100 cells was determined. Also here, concentrations of AmB (0.156 *μ*M) and DDC (1.25 mM) were used that had no or limited effect on the viability of planktonic *C. albicans *cells. The percentage of viable *C. albicans *planktonic cells after treatment with or without AmB in presence or absence of DDC is shown in Figure 3. Combined treatment of AmB and DDC resulted in an 8-fold reduction of the percentage of viable *C. albicans *cells compared to planktonic cells treated with AmB only. The percentage of viable planktonic cells after combined treatment of AmB and DDC (9 ± 1%) was significantly reduced (*P* < 0.05) compared to treatment with AmB (77 ± 20%) or DDC (47 ± 11%) alone (Figure 3). This result shows that DDC-dependent potentiation of the activity of AmB is not biofilm specific, as DDC also potentiates the activity of AmB against planktonic cells, albeit to a lesser extent.

### 3.4. ATM, a Clinical Used Copper Chelator, Increases the Activity of AmB against *C. albicans *Biofilm Cells

**Figure 4:**
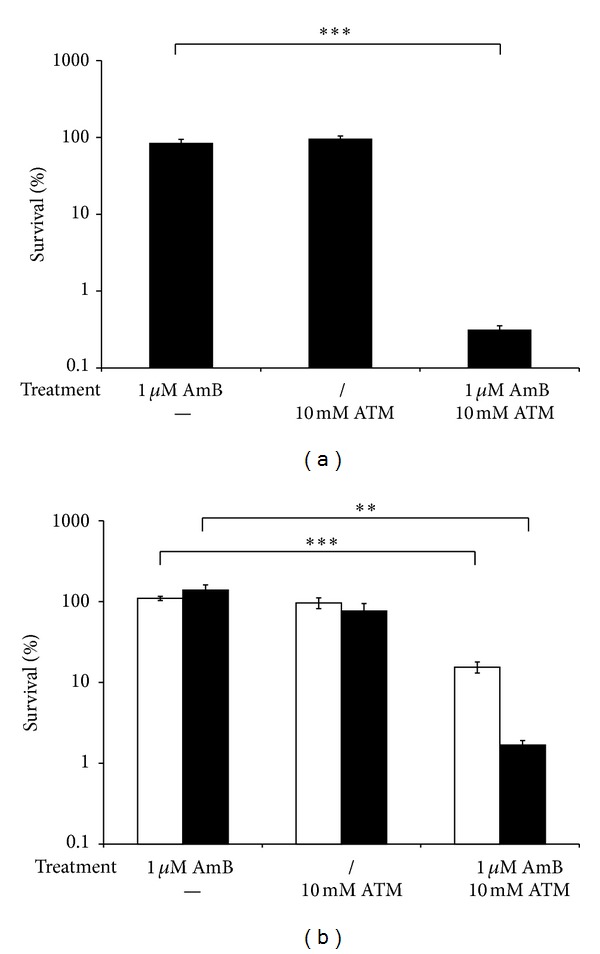
Effect of ATM on AmB-tolerant cells in biofilms of *C. albicans* CA-IF100 (a) and clinical isolates F17 and G6 (b). *C. albicans* biofilms were treated without (/) or with 1 *μ*M AmB in the absence (−) or presence of 10 mM ATM. Afterwards, biofilms were washed with PBS and survival of *Candida *cells was determined by plating the biofilm cells on YPD plates. The AmB-tolerant fraction in presence or absence of 10 mM DDC was determined relative to DMSO treatment. Data represent the mean and SEM for one representative experiment out of two, each consisting of triplicate measurements. (a) CA-IF100 and (b) clinical isolates F17 (white bars) and G6 (black bars). **P < 0.01; ***P < 0.001.

As DDC is associated with neurotoxicity [24], which might limit its clinical potential, we also determined the effect of ATM, a therapeutically used copper chelator [17, 18], on the antibiofilm activity of AmB. Coincubation of *C. albicans *CA_IF-100 biofilms with 1 *μ*M AmB and 10 mM ATM led to an approximately 300-fold reduction of viable biofilm cells, resulting in only 0.32 ± 0.04% (*P* < 0.001) of viable biofilm cells, compared to AmB treatment alone. Treatment of *C. albicans *biofilms with 10 mM ATM alone did not result in a significant reduction of the viable biofilm cells (Figure 4(a)). In addition, the percentage of viable *C. albicans *cells of two clinical isolates, F17 and G6, was also determined after treatment without or with 1 *μ*M AmB in the presence or absence of 10 mM ATM (Figure 4(b)). Co-incubation of F17 or G6 biofilms with 1 *μ*M AmB and 10 mM ATM resulted in a 7- or 80-fold significant reduction of viable biofilm cells, respectively, as compared to AmB treatment alone (Figure 4(b)). Treatment of biofilms of F17 or G6 with 1 *μ*M AmB and 10 mM ATM resulted in 15.47 ± 2.40% (*P* < 0.001) or 1.68 ± 0.22% (*P* < 0.01) viable biofilm cells, respectively, whereas treatment of biofilms of F17 or G6 with 1 *μ*M AmB alone had no effect on the viability of these biofilm cells. Treatment of these biofilms with 10 mM ATM alone resulted in no significant reduction of the viable biofilm cells (Figure 4(b)). These results indicate that ATM also increases the activity of AmB against clinical isolates, albeit to a lesser extent compared to the wild-type CA-IF100, in contrast to DDC. This might indicate that DDC is more effective in inhibiting Sods compared to ATM. However, additional studies are necessary to investigate this further.

### 3.5. Treatment with DDC and ATM Increases Endogenous Reactive Oxygen Species Levels in AmB-Treated Biofilms

**Figure 5:**
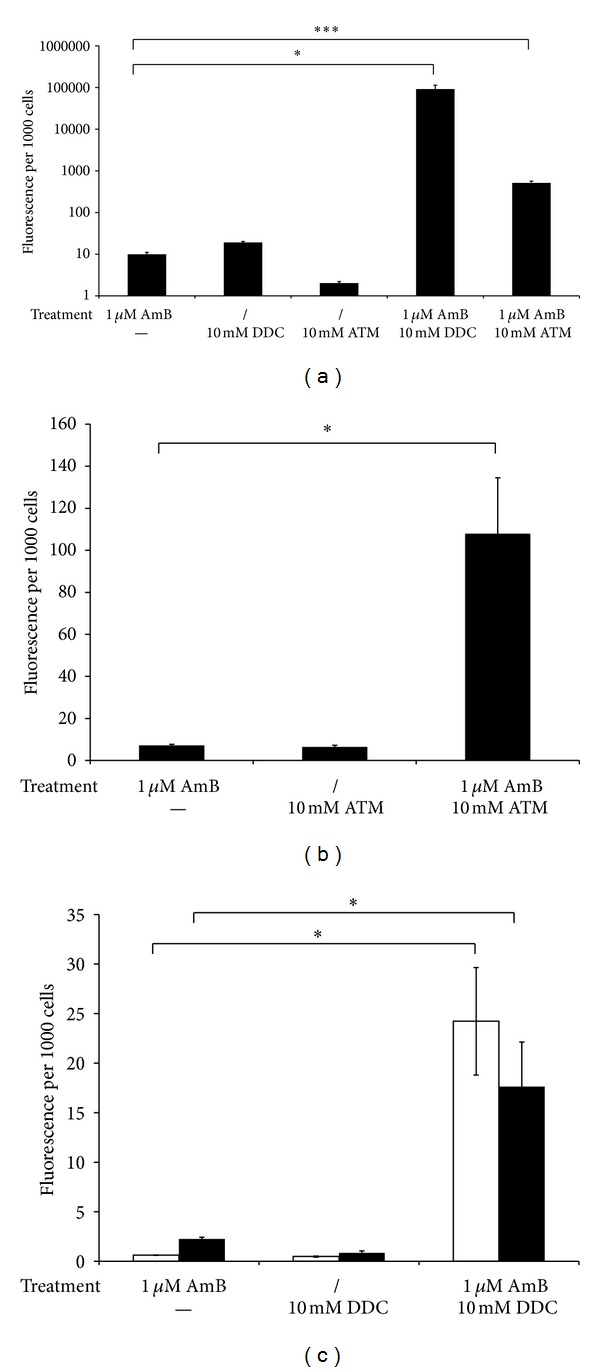
Effect of DDC and ATM on ROS levels in *C. albicans *biofilms. (a) *C. albicans *CA-IF100 biofilms were treated with or without (/) 1 *μ*M AmB in presence or absence (−) of 10 mM DDC or 10 mM ATM. Biofilms were washed with PBS and a sample was taken for CFU determination. Afterwards, 10 *μ*M DCFDA was added. Fluorescence was normalized to the number of CFUs after treatment. (b) and (c). Effect of DDC on peroxide levels on *C. albicans *biofilm cells.* C. albicans* biofilms were treated with or without (/) 1 *μ*M AmB in presence or absence (−) of 10 mM DDC. Biofilms were washed with PBS and a sample was taken for CFU determination. Afterwards, 20 *μ*M DHE was added. Fluorescence was normalized to the number of CFUs after treatment. (b) CA-IF100, (c) F17 (white bars), and G6 (black bars). Data represent the mean and SEM for one representative experiment out of two, each consisting of triplicate measurements. *P < 0.05, ***P < 0.001.

To determine if treatment with the Sod inhibitors DDC or ATM enhances endogenous reactive oxygen species (ROS) levels in AmB-treated biofilms, the accumulation of ROS was quantified using 2′,7′-dichlorodihydrofluorescein diacetate (DCFHDA) or dihydroethidium (DHE). DCF, the conversion product of DCFHDA, indicates the presence of several types of ROS, including hydrogen peroxide and peroxyl radicals, whereas DHE is a specific superoxide detection reagent [25]. In a first series of experiments, we used DCFHDA as detection reagent. Coincubation of CA-IF100 biofilms with AmB and DDC resulted in significantly increased endogenous ROS levels in *C. albicans* biofilm cells as compared to AmB or DDC treatment alone. CA-IF100 biofilms, treated with a combination of 1 *μ*M AmB and 10 mM DDC and incubated with H2DCFA, resulted in an approximately 10,000-fold increase of endogenous ROS levels (*P* < 0.05), compared to biofilms treated with AmB or DDC alone (Figure 5(a)). Also coincubation of *C. albicans* biofilms with 1 *μ*M AmB and 10 mM ATM resulted in an approximately 50-fold increase of endogenous ROS levels in *C. albicans* biofilm cells (Figure 5(a)) (*P* < 0.001), indicating again that DDC seems more effective in inhibiting Sods compared to ATM. As Sods convert superoxide to hydrogen peroxide, we have set up additional experiments in which we specifically monitored superoxide accumulation, using DHE staining, in *C. albicans* CA-IF100, G6, and F17 biofilms cells upon various treatments. Co-incubation of CA-IF100 biofilm cells with AmB and DDC resulted in an approximately 15-fold increased superoxide accumulation (*P* < 0.05), compared to biofilms treated with AmB or DDC alone (Figure 5(b)). Moreover, also biofilms of clinical isolates F17 and G6, treated with AmB and DDC, accumulated, respectively, 40- or 8-fold more superoxide compared to F17 or G6 biofilms treated with AmB alone (*P* < 0.05) (Figure 5(c)). These results show that inhibition of Sod activity by DDC in the presence of AmB results in a significantly increased superoxide accumulation. Hence, it seems that AmB specifically induces superoxide as a means to kill fungal cells, including biofilm cells.

## 4. Conclusions

All above data indicate that, in *C. albicans *biofilm cells, Sods are not only involved in protection of *C. albicans* biofilms to miconazole [16] but also to AmB, probably via detoxification of AmB-induced superoxide. 

These results are in line with results of Seneviratne and coworkers [26]. They demonstrated that *C. albicans* biofilm formation is associated with increased antioxidative capacities. Several proteins involved in oxidative stress defenses, including thioredoxin peroxidase and alkyl hydroperoxide reductase, are upregulated in biofilms, which may contribute to the higher resistance to ROS-inducing antifungals like AmB and miconazole [25]. In addition, several reports document the possibility of enhancing the fungicidal activity of ROS-inducing antifungals by targeting the oxidative stress response system of fungi. For example, Kim and coworkers demonstrated that different redox-potent chemosensitizing agents like natural dihydroxybenzaldehydes, thymol, or salicylaldehyde could enhance the antifungal activity of different ROS-inducing antifungals [27–29]. One of their studies specifically demonstrates that chemically targeting the oxidative stress response system of fungi effectively augments antimycotic potency of AmB [28]. Based on our data, it seems that Sod inhibitors can reduce the antioxidative capacities of *C. albicans* biofilm cells, resulting in increased efficacy of ROS-inducing antifungals. In a report of Walker and coworkers, it was demonstrated that the combination of DDC and AmB is effective in treating systemic *Candida *infections [30]. We now demonstrated that the combination of DDC and AmB displayed potent *in vitro* activity against biofilms of various *C. albicans* strains, including AmB-tolerant clinical isolates. However, as DDC is associated with neurotoxicity, ATM or other nontoxic and specific Sod inhibitors might lead to a novel antibiofilm combination therapy, consisting of a ROS-inducing antifungal with such inhibitor.
